# We’ll Meet Again

**Published:** 1855-03

**Authors:** 


### WE'LL MEET AGAIN.

BY OUR SISTER IN HEAVEN.*

How earthly flowers will fade !
  How earthly hopes prove vain ;
And oh, that promise, how divine,
  That friends shall meet again.

My thoughts of house and home,
  They thrill my heart with pain:
Ah ! broken, scattered family,
  Shall we not meet again ?

In that blest world above,
  Be cleared from every stain,
And be a family with God;
  O, yes, we'll meet again.

AMELIA.

* Written January, 1849; died July 16th, same year.

